# Frontal Eye Field in the Precentral Sulcus: A Direct Electrical Cortical Stimulation Study With Stereo‐EEG Electrodes

**DOI:** 10.1002/brb3.70537

**Published:** 2025-05-13

**Authors:** Yang Jin, Kaihui Li, Xining Liu, Fang Zhang, Xiao Wang, Lingxia Fei, Qinghua Tan, Danfang Li, Xiaobo Wang, Genbo Wang, Junxi Chen, Xiangshu Hu

**Affiliations:** ^1^ Department of Neurosurgery The Affiliated Guangdong Second Provincial General Hospital of Jinan University Guangzhou China; ^2^ Department of Epilepsy Center Guangdong Sanjiu Brain Hospital Guangzhou China; ^3^ Department of Neurosurgery The First Affiliated Hospital of Jinan University Guangzhou China; ^4^ Department of Neurology, Institute of Neuroscience, Key Laboratory of Neurogenetics and Channelopathies of Guangdong Province and the Ministry of Education of China The Second Affiliated Hospital, Guangzhou Medical University Guangzhou China

**Keywords:** electrical cortical stimulation | frontal eye field | precentral sulcus | seizure onset zone

## Abstract

**Background:**

This study aims to explore the stimulation‐induced eye movements (EMs) from various branches of the precentral sulcus (PrCS) and to learn if the epileptogenic area influences EMs effects.

**Methods:**

The high‐frequency direct electrical stimulation mapping reports were completed from patients with SEEG exploration. The counts of gender, the distribution of EMs effects sites, the seizure onset age, course of epilepsy, age of stimulation, and stimulation intensity were analyzed. The correlation factors of the EMs positive cases were studied through the multivariate regression method.

**Results:**

The PrCS through 778 contacts of 77 patients was studied from July 2015 to December 2020. A total of 18 patients showed induced EMs in the PrCS. The average stimulation intensity was 22.0 ± 1.3 mA. In positive cases, 279 contacts were explored on the PrCS, and 24.51% were positive. Overall, 60.32% sites were in the superior PrCS (SP), and 39.68% sites were in the inferior PrCS (IP). The SP was more likely to have a stimulating response to EMs than the IP (*p* = 0.017). The major EM effect was deviation, with a total of 39.68% positive sites in 11 cases, namely, 16 sites in the SP and 9 sites in the IP. Multiple regression analysis indicated that the positive EMs cases were only correlated with whether seizure onset zone (SOZ) was located on prCS (*p* = 0.004), but not with age of onset, duration, frequency of seizures, preoperative intelligence quotient (IQ), and age of stimulation.

**Conclusion:**

Deviation was the major type of positive EMs by directly stimulating the prCS. The SP was more sensitive to stimulation than the inferior branch was. The positive EM's chance showed a certain correlation with whether the prCS was inside the SOZ or not.

## Graphical abstract

Ocular movements included deviation, versive movement, and eyelid movements. Deviation was the major type of positive eye movements by directly stimulating the precentral sulcus (prCS). The superior precentral sulcus was more sensitive to stimulation than the inferior branch was.

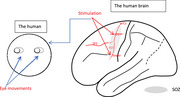

**Abbreviations**
ACC: anterior cingular cortex
ADS: after discharges
c: caudal part of
CS: central sulcus
d: dorsal part of
EA: epileptogenic area
EMs: eye movements
ESM: electrical cortical stimulation mapping
FCD: focal cortical dysplasia
FEF: frontal eye field
HE: horizontal branch of the inferior precentral sulcus
IP: inferior precentral sulcus
j: junction of
L: left
OFC: orbital frontal cortex
OP: operculum
PCC: posterior cingular cortex
PMV: ventral premotor cortex
postCG: posterior central gyrus
PrCS: precentral sulcus
pre‐SMA: pre‐supplementary sensorimotor area
Pt: patient
R: right
SEEG: stereo‐electroencephalography
SFG: superior frontal gyrus
SFS: superior frontal sulcus
SIS: stimulation‐induced seizures
SMA: supplementary sensorimotor area
SMG: super‐marginal gyrus
SOZ: seizure onset zone
SP: superior precentral sulcus
v: ventral part of

## 1 Introduction

Frontal eye field (FEF) is one of the important eye movement (EM) control cortices (Gaymard et al. 1998; Anderson et al. 1994; Pierrot‐Deseilligny et al. 2005). Comparative anatomical and functional MRI studies have confirmed that the human FEF is located in the precentral sulcus (prCS) on the caudal part of the lateral frontal lobe (Vernet et al. 2014; Paus 1996) and is divided into upper and lower parts (Derrfuss et al. 2011; Amiez and Petrides 2009; Schmitt et al. 2005). The ventral branch of superior prCS (SPv), especially the junction of superior frontal sulcus (SFS) and prCS, is the classic core region of the FEF. As Petrides reviewed, the electrical cortical stimulation mapping (ESM) studies in the human brain showed the FEF lying in the caudal part of the middle frontal gyrus close to and within the ventral branch of the superior prCS (SP) (Amiez and Petrides 2009). ESM results mainly depended on the electrical current used and the relatively limited region explored by electrode implanted. By subdural grid electrodes, EMs were induced from a small area in the cortex anterior to the junction of the SFS with the prCS in three cases (Blanke et al. 2000). However, in other 12 cases, 2 different EMs areas were elicited from human FEFs by stereotactic electrodes. One area was located at the intersection of the SFS with the fundus of the superior portion of the prCS, and the other lied closely to the surface of the prCS. SEEG takes an advantage in the study of stimulation effects in the bottom cortex of the prCS (Lobel et al. 2001). In the limited case studies of human FEF stimulation, the effect of epileptogenic zone on stimulation response of fronto‐ocular region has not been mentioned (Derrfuss et al. 2011; Blanke et al. 2000; Lobel et al. 2001; Kimura et al. 2021; Godoy et al. 1990). As ESM is based on intracranial electrode implantation in patients with epilepsy, stimulation effects may be affected by many factors associated with epilepsy, such as age of seizure onset, duration, nature and location of lesions, and age of stimulation, which may impact on brain functional remodeling in turn (Arya et al. 2020; Batschelett et al. 2021; Hyslop and Duchowny 2019). These factors have infrequently been mentioned in previous cortical stimulation studies in frontal cortex (Blanke et al. 2000; Lobel et al. 2001; Kimura et al. 2021). Therefore, we performed direct electrical cortical stimulation on the prCS in drug‐resistant epilepsy patients through stereotactic electrodes. It aims (1) to explore the localization and characters of induced EMs by stimulating the prCS and (2) to find out the correlation between the high‐frequency stimulation EMs responses in this region and the location of the epileptogenic area (EA).

## 2 Materials and Methods

### 2.1 Patients

The samples were collected from patients with completed reports, who underwent SEEG recording and high‐frequency ESM from July 2015 to December 2020 in Guangdong Sanjiu Brain Hospital.

Inclusion criteria: (1) Patients with EMs effects induced by stimulating the prCS in records were found, (2) positive sites were further checked to confirm on the wall of the prCS. (3) Those with complete clinical data. Exclusion criteria: (1) Those with reports poorly described about EMs, (2) stimulation structures were unconfirmed, and (3) those who are unwilling to participate in this research.

All patients underwent a comprehensive non‐invasive presurgical evaluation, including history, telemetry, seizure semiology, intelligence quotient (IQ) score, brain CT, MR, and FDG‐PET. Electrode implantation strategies were designed on the basis of the working hypothesis of epileptogenic zone after discussion and decision‐making by multiple disciplines. The implantation was guided by using a frameless stereotactic assisted localization system. After intracranial EEG information about seizure onset zone (SOZ) was analyzed, direct stimulating mapping was performed. All patients signed informed consent.

### 2.2 Electrical Cortical Stimulation Mapping

The examination was performed by Neurofax EEG‐1200 with a JE‐120 amplifier, an MS‐120‐EEG cortical stimulator, and a Nihon Kohden PE‐210 software stimulator switch box (Nihon Kohden, Tokyo, Japan). The procedure was extraoperatively performed 1–3 days after anti‐seizure medication was resumed and SOZ had also been ascertained. Stimulation protocols were 50 Hz, biphasic, 300 µs for pulse duration, and trains with 3–5 s. We started to stimulate from 0.1 mA, 0.1 to 1 mA step, maximum to 5 mA, until functional response occurred, after discharges (ADS) elicited or seizure induced. The same electrode was performed in pairs from the deepest to the most superficial one with two adjacent contacts (Trébuchon and Chauvel 2016; So 2018). The positive effect was evaluated by two senior neurophysiologists during the procedure. When a dispute occurred, a consensus was reached, and a report was written after review.

### 2.3 Stimulated EMs Effects

Ocular movements included (1) deviation: contralateral eyes gaze without accompanying head turning; (2) versive movement: contralateral forced tonic turning of eyes and head; (3) eyelids+: eyelids movements, like fast blinking, with or without facial movement; (4) deviation + E (D + E): contralateral gaze with eyelids movements; (5) deviation + flip angle (FA) (D + FA): contralateral gaze with facial or arm tonic movement; and (6) SIS: stimulation‐induced seizures (Tanner and Lüders 2008).

### 2.4 PrCS Segment Definition

The structure of the prCS was categorized into six parts. (1) SPd: the SP dorsal ramus; (2) SPj: intersection with the SFS, (3) SPv: ventral ramus of the SP, (4) IPd: the inferior prCS (IP) dorsal branch, (5) HE: horizontal branch of the IP, (6) IPv: ventral branch of the IP (Amiez et al. 2006; Germann et al. 2005).

### 2.5 Epiletogenic Area Classification

The SOZ was fulfilled by the following criteria: the electrode site where the initial ictal electrical changes met the EEG onset pattern (Lagarde et al. 2018). According to the SOZ, the EA was classified into (1) Group 1 (prCS group): There was at least one contact on the prCS wall within the SOZ region; (2) Group 2 (non‐prCS group): None of the SOZ contacts were on the wall of the prCS.

### 2.6 Localization Method to the Brain Structure Explored by SEEG

#### 2.6.1 Protocol Acquisition

MRI: Using 3T scanners, the scan was performed using a three‐dimensional brain volume imaging (3D‐BRAVO) sequence with echo time (TE) of 3.5 s, repetition time (TR) of 8.8 s, FA of 13°, and voxel size of 1 mm × 1 mm × 1 mm (no interslice gap). CT images were acquired with slice interval of 0.5 mm, slice thickness of 1.0 mm, and resolution of 512 × 512.

#### 2.6.2 Individual Reconstruction

The original DICOM data were converted to NIfTI files, CT/MR registration was performed using the elastix module (Klein et al. 2009) in 3D Slicer 4.11 software (https://www.slicer.org/), and then electrode contacts were reconstructed through the SEEG Assistant plugin (Narizzano et al. 2017; Arnulfo et al. 2015) (https://mnarizzano.github.io/SEEGA/). Two experienced epileptologists verified the location of electrode contacts, which is the SFS and the prCS, respectively. And then the previously placed electrode contacts were retained for further procedures.

#### 2.6.3 Spatial Normalization

The FreeSurfer 7.0 software (https://surfer.nmr.mgh.harvard.edu/) was used for cortical segmentation and brain surface calculation by the Colin 27 2008 template file. The results were then imported into 3D Slicer to reconstruct surfaces of brain, the prCS, and the SFS. The nonlinear registration method was applied to analyze the individual patient data through the Colin 27 template to obtain the three‐dimensional spatial projection, and SEEG electrode contacts were coordinated in the template.

### 2.7 Statistical Analysis

All cases were divided into two groups: One was the EMs positive (EMs+) group, and the other was the EMs negative (EMs−) group. The following variables were collected. Continuous variables, such as age of onset, disease duration, stimulation age, IQ score, numbers of electrode contacts on prCS, and stimulation intensity, were applied by the mean ± standard deviation. Categorical variables such as sex, seizure frequency, etiology, and EA location were represented by Arabic numerals. *T*‐test was used to compare the counting data, and Chi‐square test was used to compare the categorical data between two groups. The variables with *p* value less than 0.1 were further used to analyze the related factors of the EM positive group by multiple regression analysis.

## 3 Results

From July 2015 to December 2020, a total of 77 SEEG patients were explored on the prCS. Seventy cases were identified with single SOZ. In the EMs positive group (Table 1), there were 13 males and 5 females, and 10 were explored on the left side and 8 on the right, being with 63 positive sites in total. Brain MR images showed MCD in 16 cases, with pathological confirmation in 6 cases and other etiologies in 3 cases. The age of receiving ESM ranged from 7 to 29 years old; the average age was 17.06 ± 6.90 years; and the mean duration of epilepsy was 8.70 ± 8.29 years. The average stimulation intensity was 2.0 ± 1.3 mA; the mean stimulation time was 3.08 ± 0.38 s.

**TABLE 1:** Demography and clinical data of 18 patients with positive eye movements (EMs) effects from directed stimulation mapping.

Pt	Sex	Age	Seizure onset age (Y)	Epilepsy duration (Y)	Etiology		Side	EA	EA group	Sites in PrCS (*n*)	EMs sites (*n*)	EMs effect^a^	Stimulation intensity range (mA)
					MR	Pathology							
1	M	18	16	2	FCD	FCDIIa	R	SFG	Non‐PrCS	5	3	1	0.8–1.6
2	F	12	9	3	Encephalomalacia	Encephalomalacia	L	SMG	Non‐PrCS	6	4	1,3	0.8–2.4
3	M	17	16.5	0.5	Encephalomalacia	Non	L	PostCG + OP	Non‐PrCS	19	2	5	2.4–2.6
4	M	27	10	17	FCD	FCDIIb	L	SPv	PrCS	20	3	1,4,5	1.0–5.0
5	M	20	18	2	FCD	Neurogliosis	L	PCC + SMG	Non‐PrCS	9	2	1	1.6–2.2
6	M	15	5	10	FCD	m‐MCD	L	SFS + SPv	PrCS	11	2	3	4.0–5.0
7	M	28	24	4	MCD	Non	R	SMA	Non‐PrCS	15	4	1	0.8–5.0
8	M	19	17	2	FCD	Non	L	IPv	PrCS	33	13	1,2,3,4	1.0–5.0
9	M	23	3	20	FCD	m‐MCD	L	OFC + HE + IPd + IPv	PrCS	18	3	1,5	1.0–1.8
10	M	19	5	14	FCD	Non	R	PrCG + CS	Non‐PrCS	20	2	1,6	1.0–1.2
11	M	29	5	24	FCD	Non	R	SMA	Non‐PrCS	12	2	4,5	0.8–1.4
12	M	10	3	7	FCD	FCDIIb	L	HE	PrCS	8	2	1,3	3.0
13	F	7	5	2	FCD	Non	R	SPv + PrCG	PrCS	17	3	3,6	1.0–1.8
14	F	13	8	5	FCD	Non	R	SPv	PrCS	24	8	1,2,5	1.0–5.0
15	M	9	4	5	FCD	Non	L	SPv	PrCS	33	1	6	1.0
16	F	21	14	7	FCD	Non	R	PrCG	Non‐PrCS	6	4	2,4	0.8–1.0
17	F	13	7	6	FCD	FCDIIa	L	SPj + SFSc	PrCS	6	4	2,5	0.5–1.0
18	M	7	4	3	FCD	Non	R	PreSMA + ACC	Non‐PrCS	17	1	1	2.0

^a^ EMs effect: 1, deviation; 2, versive movement; 3, eyelids+; 4, deviation + E; 5, deviation + FA; 6, SIS.

### 3.1 Location of EMs Effects and Stimulation Parameters

**FIGURE 1:**
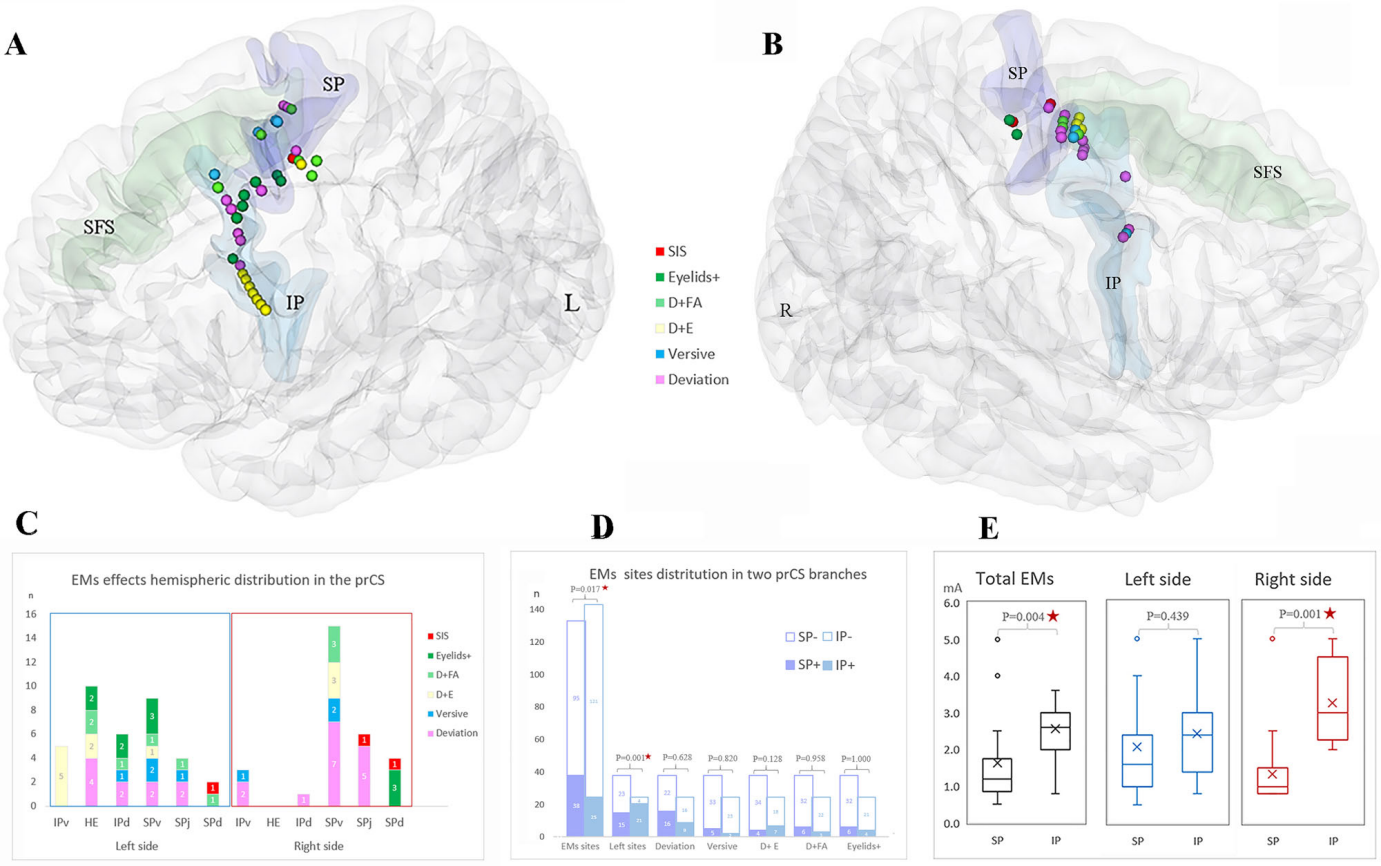
General view of induced eye movements effects in the precentral sulcus. The 3D brain maps of A and B represent the left and right hemispheres separately. Charts C and D are the hemispheric distribution of the number of positive sites in different branches of the precentral sulcus for each ocular movement effect. Chart E shows stimulation intensities.

Among all EM+ cases, seven cases showed one type of EMs effects, eight cases showed two types, and three cases showed three types or more. The minimum stimulation intensity for different patients ranged from 0.8 to 4.0 mA, with an average of 1.36 ± 0.92 mA. Sites of positive effect were on the SP in 13 cases and on the IP in 5 cases, with a tendency of statistical difference (*p* = 0.079). Among all those positive EMs sites, 25 sites were elicited with deviation, which was the most constant effect (25/63%, 39.68%). Seven sites were versive movement, 11 were deviation with eyelids movement, 9 were deviation with facial upper limb movement, and 10 were eyelids movement. A total of 38 positive sites were in the SP (38/63, 60.32%), with all 5 types of EMs effects recorded in its ventral branch. The other 25 positive sites were located in the IP (25/63, 39.68%). The SP was more likely to show ocular responses than the IP (*p* = 0.017) (Figure 1D). In addition, EMs sites distribution in different segments of the prCS in two hemispheres were significantly different (*p* = 0.001) (Figure 1D). Specifically, deviation with eyelids movement was more induced in the IP than it was in the SP, and other EMs effects showed the opposite distribution characteristics (Figure 1D). The stimulation intensity of the SP was significantly lower than that of the IP (*p* = 0.004) (Figure 1E), and a similar pattern occurred in the right hemisphere (*p* = 0.001) (Figure 1E).

### 3.2 Analysis of Stimulated Deviation Effect

**FIGURE 2:**
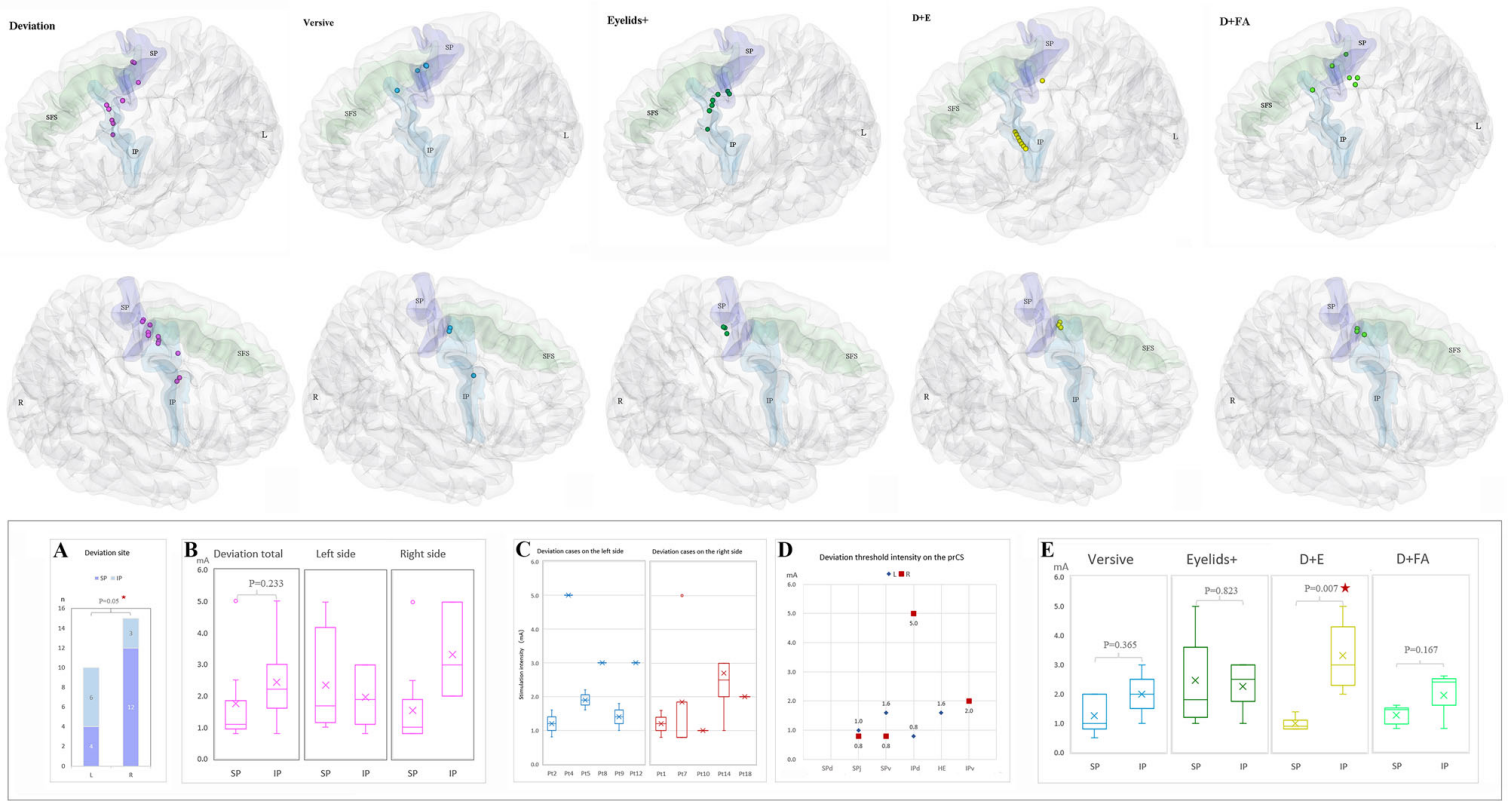
The hemispheric EMs distribution maps of stimulation sites and the stimulating intensity charts. The 3D brain maps of the upper row show the five different EMs effects distribution on the left sites. The maps of the lower row exhibit the EMs sites on the right side. Chart a represents the location analysis for the deviation effect. Charts b‐d show the intensity of deviation in various conditions. Chart e exhibits the intensity of the other EMs effects.

There were 11 patients with 25 sites elicited deviation effect. Six cases with 10 sites were in the left hemisphere, and 5 cases with 15 sites were on the right side. Among these sites, 16 sites were on the anterior or posterior bank of SP, 11 on the region of SPj and 9 on the SPv segment, respectively, and none on the SPd branch (Figure 2A). Only one right SPv site showed contralateral oblique upward gaze. The deviation effect could also be observed in branches of the IPd and the IPv. Compared to the hemispheric distribution of stimulation sites, there was a significant statistical difference between the right SP and the left one, *p* = 0.05 (Figure 2A). The distribution of this effect varied greatly among individuals. There were five cases with deviation only induced in the SP and three cases merely in the IP. In the other three patients, this effect was elicited both in the SP and the IP, and the threshold stimulation occurred in the SP in two cases and in the IP in one case. The stimulation intensity varied from 0.8 to 5.0 mA, showing a discrete trend among different individuals (Figure 2C,D). The average stimulation intensity of the SP and the IP was 1.74 ± 1.36 and 2.42 + 1.26 mA, respectively, with no statistical difference between them (*p* = 0.233) (Figure 2B).

### 3.3 EMs Effects and EAs

In all cases with electrodes covering the prCS, the characteristics of EMs+ and EMs− cases were compared (Table 2‐1 and 2‐2). There were no significant differences in gender (*p* = 0.689), seizure onset age (*p* = 0.931), course of disease (*p* = 0.970), seizure frequency (*p* = 0.468, 0.265, 0.897), etiology (*p* = 0.570), and preoperative IQ (*p* = 0.644, 0.866, 0.273) between the two groups. There was no statistically significant difference between the two groups either when comparing certain locational characteristics of the EA, such as hemispheric lateralization (*p* = 0.875) and involvement of the frontal lobe (*p* = 0.570). However, regarding whether the SOZ was located on the prCS and the number of electrode contacts covering this region, a significant difference was observed between the two groups (*p* = 0.000, 0.009). Multiple regression analysis indicated that the EM+ group was solely associated with whether the SOZ was located on the prCS (*p* = 0.004).

**TABLE 2‐1:** Factors related to eye movements (EMs) cases in Step 1 regression analysis.

Characters			Total	EMs+ group	EMs− group	*p* value
Patients (*n*, %)			70	18 (25.7)	52 (74.3)	
Sex (*n*, %)	Male:Female		53:17	13 (24.5)	40 (76.9)	0.689
Sz onset age (y)			8.4 ± 7.9	8.3 ± 5.4	8.5 ± 10.5	0.931
Epilepsy duration (y)			8.6 ± 7.4	8.7 ± 8.3	8.6 ± 7.2	0.970
Stimulation age (y)			17.1 ± 10.5	17.1 ± 6.9	17.1 ± 11.5	0.989
Sz frequency (*n*, %)	Daily		36	7 (19.4)	29 (80.6)	0.468
	Weekly		16	5 (31.3)	11 (68.7)	0.265
	Monthly		18	6 (33.3)	12 (66.7)	0.897
Etiology (*n*, %)	MCD		55	15 (27.3)	40 (72.7)	0.570
	nonMCD		15	3 (20.0)	12 (80.0)	
IQ (score, *n* = 58)	FIQ		85 ± 21.0	87 ± 24.6	84 ± 19.7	0.644
	VIQ		84 ± 21.0	84 ± 24.7	83 ± 19.6	0.866
	PIQ		89 ± 19.6	93 ± 21.2	87 ± 18.7	0.273
SOZ hemisphere (*n*, %)	Left		40	10 (25.0)	30 (75.0)	0.875
	Right		30	8 (26.7)	22 (73.3)	
SOZ lobe (*n*, %)	Frontal		56	16 (28.6)	40 (71.4)	0.570
	nonFrontal		14	2 (14.3)	12 (85.7)	
SOZ in frontal lobe (*n*, %)		prCS	13	9 (69.2)	4 (30.8)	**0.000**
		Non‐prCS	43	7 (16.3)	36 (83.7)	
PrCS electrode contacts (*n*)	SP/Mean value per case		379/5.4	149/8.3	230/4.4	0.060
	IP/Mean value per case		399/5.7	130/7.2	269/5.2	0.111
	Total/Mean value per case		778/11.1	279/15.5	499/9.6	**0.009**

*Note*: Bold value significant *P* value lower than 0.05.

**TABLE 2‐2:** Factors related to eye movements (EMs) cases in Step 2 regression analysis.

								95% confidence interval for exp. (B)	
EMs groups		B	Std. error	Wald	df	Sig.	Exp. (B)	Lower bound	Upper bound
EMs+	Intercept	1.916	1.561	1.506	1	0.220			
	Concact in prCS	0.106	0.073	2.117	1	0.146	1.111	0.964	1.281
	Concact in SP	−0.060	0.078	0.583	1	0.445	0.942	0.808	1.098
	SOZ in PrCS (1 = in, 0 = out)	−2.214	0.769	8.283	1	**0.004**	0.109	0.024	0.493

*Note*: The reference category is EMs−. Bold value significant *P* value lower than 0.05.

**FIGURE 3:**
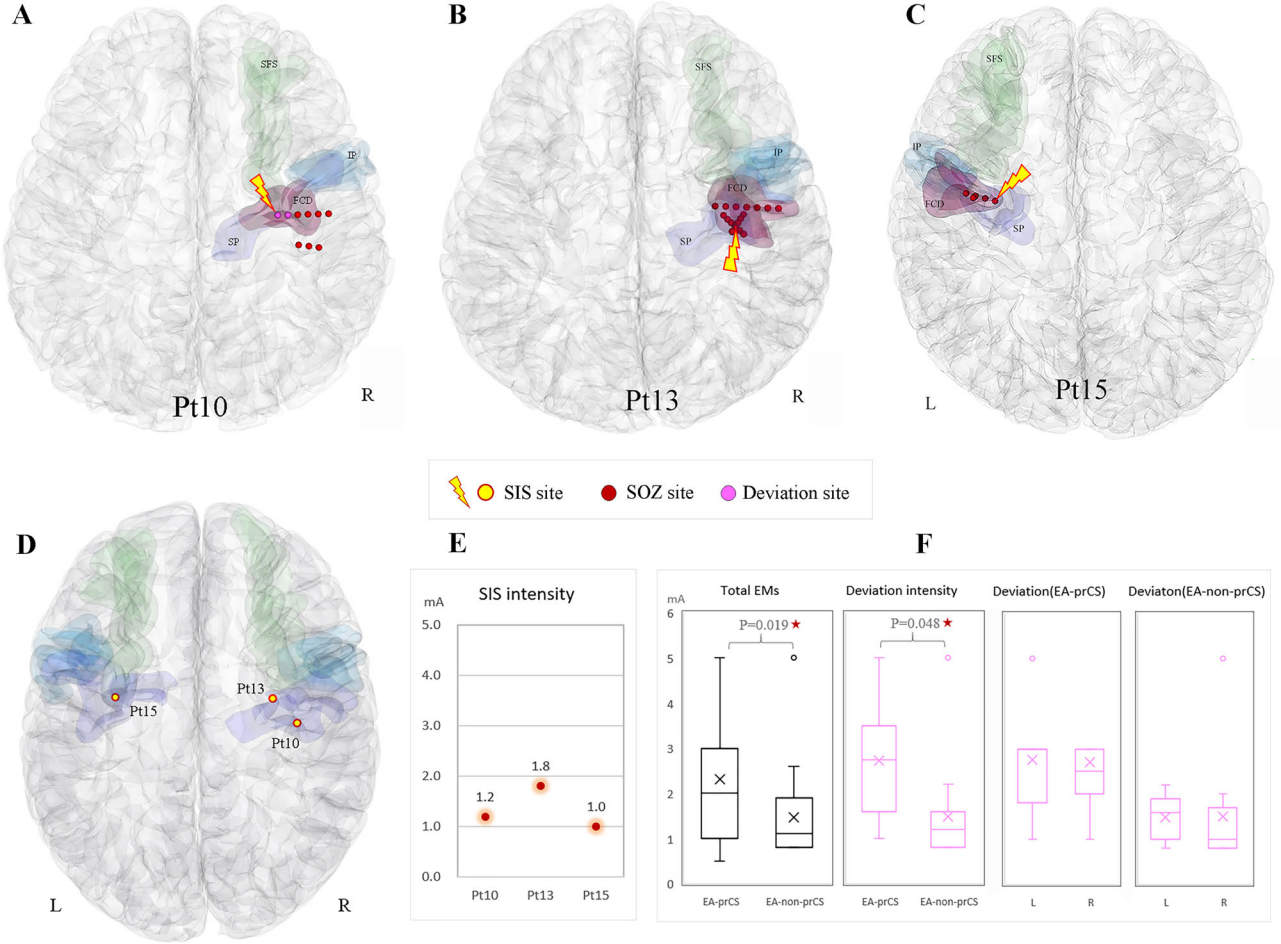
The relationship between SIS site, SOZ and FCD lesions in 3D brain maps (A‐D). In panel A, the purple‐red SIS site represents induced deviation at 1.0mA. Chart E shows the SIS intensity. Chart F represents stimulation intensities according to the EA grouping.

The EA of EM+ group was further analyzed. Nine cases were in the prCS group, with the EA of six cases in the SP and three cases in the IP. There were 170 contacts on the prCS in this group, 39 sites were EMs positive (22.94%), and the average stimulation intensity was 2.3 ± 1.4 mA. The other nine cases were in the non‐prCS group, with the EA of four cases in the medial frontal lobe, three cases in central regions, and two cases in the parietal lobe (Table 1). In this group, 109 contacts were on the prCS, 24 sites of them were EMs positive (22.02%), and the average stimulation intensity was 1.5 ± 1.0 mA. The mean intensity of the two subgroups was significantly different (*p* = 0.019). The deviation intensity exhibited similar characteristics (Figure 3F). Three sites of stimuli‐induced seizures from three different cases (3/18, 16.7%) were all located in the SP (Figure 3D). The SOZs of two cases (Pt13, Pt15) were located in the SPv; evoked sites were just inside the SOZ (Figure 3B,C). The other case's (Pt10) SOZ was in the central gyrus near the SPj (Figure 3A). The SIS that originated from the region inside SOZ had the similar seizure semiology as the usual one, yet the one that originated from the region outside SOZ showed a new seizure semiology. Deviation appeared to be the initial symptom in two SIS.

## 4 Discussion

### 4.1 Elicited EMs Effects by Stimulating the PrCS

Versive EM, being marked sustained lateral positioning of the eyes, called deviation in the study, was the main oculomotor effect in stimulating in the FEF region (Godoy et al. 1990). Early ESM studies by using SEEG confirmed that the intersection of the bottom of the SP and the posterior end of the SFS was one of regions of the FEF (Lobel et al. 2001). This deep region was highly sensitive to high‐frequency intracranial stimulation at a minimal intensity of 0.8 mA, much lower in this data than in others. The second oculomotor area was close to the lateral surface of the prCS (Lobel et al. 2001). This area was found to located specifically on the branches of SPv, IPd, and IPv. Anatomical landmarks of the prCS could indicate the location of FEF functional subregions, namely, saccade control cortex deep in the prCS and pursuit in the lateral prCS (Amiez et al. 2006). It is worth noting that the specific location of this effect in the prCS was different among different individuals (Blanke et al. 2000; Lobel et al. 2001; Kimura et al. 2021; Godoy et al. 1990).

Lateral gaze elicited from the prCS was occasionally accompanied by other movements, such as codirectional head turning, blinking, or contraction of upper limb or face on the same side (Lobel et al. 2001; Kimura et al. 2021). As the hand movement control cortex within the SPd was highly adjacent to the saccade control cortex, accompanying contraction of upper limb with deviation may induced from sites of the SP (Amiez and Petrides 2017). Meanwhile, inferior FEF (iFEF) may be involved both in blinking and facial movement control (Chan and Downing 2011; Willett et al. 2023), accompanied eyelids movement with deviation, or even solely eyelids movement could be elicited from sites of the IP.

Upward gaze was rarely occurred in stimulating on the prCS (Paus 1996). In our 63 positive sites, contralateral oblique upward gaze was observed from only 1 site on the right SPv. There were evidences for three‐dimensional cortical control of gaze by the FEF (Thurtell et al. 2009). When the patient looked downwards during stimulating right SP, pure upward vertical conjugate EMs were evoked with head, followed by full‐range upward gaze direction (Kaiboriboon et al. 2012). Stimulation‐evoked oblique EMs appeared when the eyes started from an eccentric position, other than the primary position (Blanke and Seeck 2003).

The laterlization of the FEF may exist. Marco's group found that the left superior FEF was close to the anterior wall of the prCS, whereas the right one was close to the posterior wall (Bedini et al. 2023). The iFEF is located only in the dorsal branch of the IP on the left side (Derrfuss et al. 2011). EMs positive sites were gathered in the IP on the left side, whereas in the SP on the right. The right SP was more responsive than the left one for deviation effect. Different methodology may be a factor to impact the FEF laterlization. The asymmetry of specific premotor‐parietal‐frontal pathway may be another factor. For example, in the dorsal premotor area, the connection between the dorsolateral prefrontal lobe and the parietal lobe was left‐sided, while the ventral premotor cortex and the angular gyrus connection was right‐sided (Tomassini et al. 2007).

In our study, the stimulation current of positive EMs effects was lower in the SP than in the IP, which had not been reported before. The deviation effect was induced in the SP by the mean intensity of 1.7 ± 1.4 mA, while that in the IP was 2.4 ± 1.3 mA. An increased intensity may expand the range of oculomotor effective areas (Amiez and Petrides 2009; Trébuchon and Chauvel 2016). The threshold intensity was lower than those reported in literatures (Schmitt et al. 2005; Blanke et al. 2000; Amiez et al. 2006), which was related to the use of different types of electrodes (Grande et al. 2020).

### 4.2 Influence of EA on Stimulation Effect of FEF

The ESM positive motor cortex is highly consistent with its functional anatomical localization (Tanner and Lüders 2008; Viganò et al. 2018). The localization may be associated with age, underlying specific pathological conditions, functional transfer, or remodeling (Batschelett et al. 2021; Hyslop and Duchowny 2019; Säisänen et al. 2020). Stimulation effects in the FEF may be affected by the ESM performing age, for both frontal saccade control and frontoparietal ocular control networks are age‐dependent (Chen and Machado 2016). In this study, the presence of the SOZ within the prCS emerged as the sole correlating factor responsible for eliciting a positive oculomotor response to prCS stimulation. Conversely, variables, such as age at onset, seizure duration, seizure frequency, etiology, age at stimulation, and IQ, did not demonstrate any association with this response.

The mean responsive threshold intensity was influenced by the age of seizure onset and the etiology of epilepsy (Hyslop and Duchowny 2019). The simulation threshold was increased with the epilepsy onset age, and it was higher in younger children and in patients with neuronal migration disorders in the paracentral region. Children with tumors, abscesses, hemangiomas, and cavernous tumors near the motor area may need greater current intensity (Arya et al. 2020). Our findings indicate that the stimulation intensity required to elicit the deviation effect was significantly greater in epileptogenic prCS sites compared to non‐epileptogenic sites. This may partially explain why there were fewer deviation sites observed in the epileptogenic region than in the non‐epileptogenic region within this study.

The structure of SIS made a great contribution to localizing the EA caused by FCD (Zhao et al. 2023). FCD associated SIS in the FEF followed this rule. Causes of SIS in the three studied cases were all FCD in frontal lobe. Induced sites were all in the SP inside FCD lesions as well; two of them were located in the SOZ, and the other one was adjacent to the SOZ. Notably, the chance of SOZ overlap in frontal SIS and spontaneous seizure may not be as high as that of medial temporal epilepsy (Kovac et al. 2014).

## 5 Limitations

This was a single‐center retrospective study, and all positive reactions were based on reports. The nature of EMs could not be analyzed in detail, and the saccade or the smooth pursuit function of the prCS could not be further located either. Due to limited sample size, the EMs effects mapping was not studied on the anterior or posterior wall of the prCS. Neither research related to the intensity of ADS was done, nor did it mention the difference between the stimulated EMs effects in the prCS and other cortical eye fields in this article.

## 6 Conclusion

Direct electrical cortical stimulation through SEEG could accurately and effectively mapping two portions of the EMs cortex in the prCS, and one medial portion was in the ventral branch of the SP, especially the area at the intersection of the SP and the caudal SFS. The other lateral portion was located in the IP three branches. Various types of oculomotor responses were elicited by direct stimulating on the prCS; deviation was the main response among them. The chance of positive EMs in this region was related to whether the prCS was inside the SOZ or not, whereas the seizure onset age, duration, seizure frequency, cause of epilepsy, or the stimulation executive age were irrelevant.

## Author Contributions

**Yang Jin**: writing–original draft, data curation, methodology, formal analysis. **Kaihui Li**: software, writing–original draft, formal analysis. **Xining Liu**: data curation, investigation. **Fang Zhang**: methodology. **Xiao Wang**: formal analysis, supervision. **Lingxia Fei**: formal analysis, supervision. **Qinghua Tan**: formal analysis, visualization. **Danfang Li**: formal analysis, visualization. **Xiaobo Wang**: resources, investigation. **Genbo Wang**: software. **Junxi Chen**: software, validation, funding acquisition. **Xiangshu Hu**: writing–review and editing, conceptualization, project administration.

### Peer Review

The peer review history for this article is available at https://publons.com/publon/10.1002/brb3.70537

## Data Availability

The data that support the findings of this study are available on request from the corresponding author. The data are not publicly available due to privacy or ethical restrictions.
